# Cardiovascular magnetic resonance features of caseous calcification of the mitral annulus

**DOI:** 10.1186/1532-429X-10-25

**Published:** 2008-05-26

**Authors:** Lorenzo Monti, Eva Renifilo, Manuel Profili, Luca Balzarini

**Affiliations:** 1 Department of Radiology, I.R.C.C.S. Istituto Clinico Humanitas, Rozzano, Milan, Italy; 2 Department of Cardiology, I.R.C.C.S. Istituto Clinico Humanitas, Rozzano, Milan, Italy; 3 Cardiovascular Magnetic Resonance, I.R.C.C.S. Istituto Clinico Humanitas, Via Manzoni 56, 20089 Rozzano (MI), Italy

## Abstract

We present two cases of caseous calcification of the mitral annulus studied by Cardiovascular Magnetic Resonance; the diagnostic feature of this rare cardiac mass are described.

## Introduction

Cardiovascular Magnetic Resonance (CMR) is unrivalled as an imaging modality for the evaluation of cardiac and pericardial masses. Caseous calcification of the mitral annulus is a rare [1-4] form of degeneration of the fibrous skeleton of the mitral annulus that should be included in the differential diagnosis of myocardial masses. Usually found in elderly patients, it's typically located in the posterior mitral annulus. We studied with CMR two cases of caseous calcification of the mitral annulus; in both cases the diagnosis was confirmed with a CT scan.

## Case report

**Figure 1:**
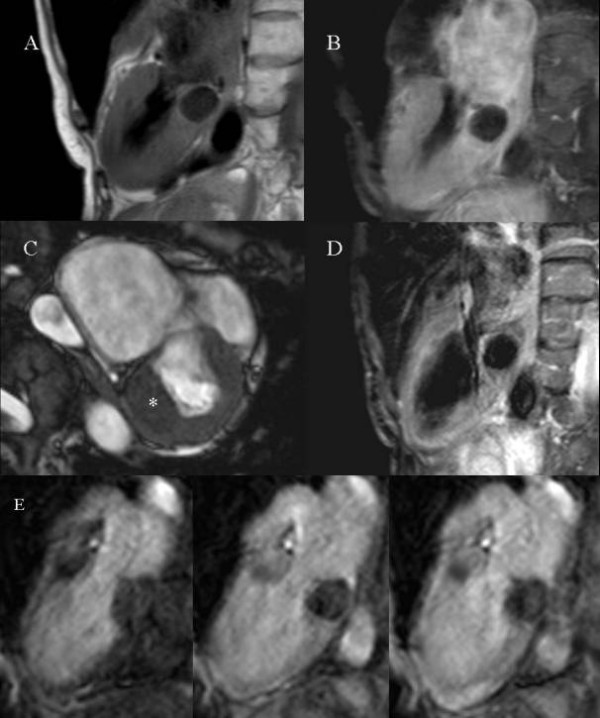
Patient 1: Panel A: T1-W turbo spin echo sequence. Panel B: STIR (Short Tau Inversion Recovery) sequence. Panel C: bSSFP sequence. Panel D: post-contrast T1-w turbo spin echo sequence. Panel E: first pass perfusion.

Patient 1. A 87-year-old woman was referred to our Hospital with suspicion of an atrial mass. A CMR study (Figure 1) showed appearances compatible with extensive caseous calcification of the posterior mitral annulus, with dimensions of 3 × 2.5 cm and a circumferential extension of about 5 cm, in the basal inferior wall of the left ventricle and bulging into the posterior left atrium, without significant mitral valve regurgitation.

**Figure 2:**
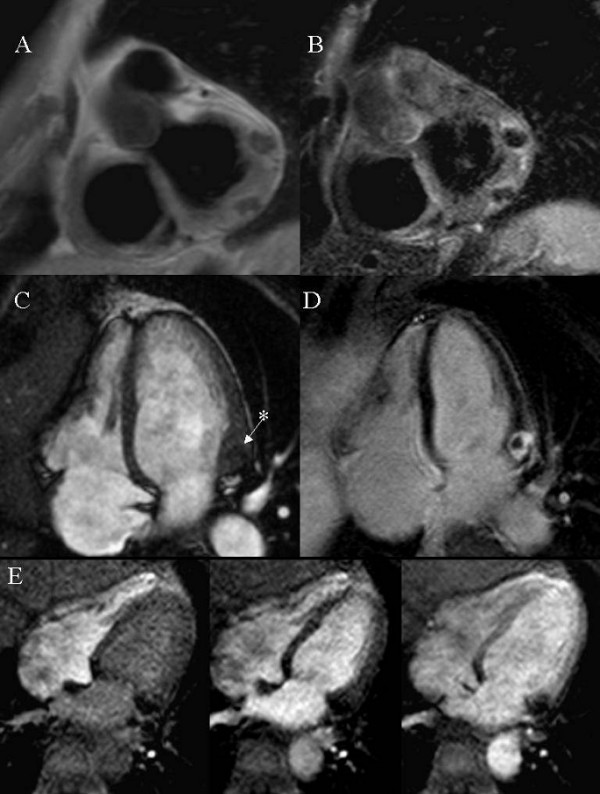
Patient 2: Panel A: T1-W turbo spin echo sequence. Panel B: STIR (Short Tau Inversion Recovery) sequence. Panel C: bSSFP sequence. Panel D: Delayed Enhancement, 10 minutes after contrast administration: an enhanced rim appears to surround a non-enhanced core. Panel E: first pass perfusion.

**Figure 3:**
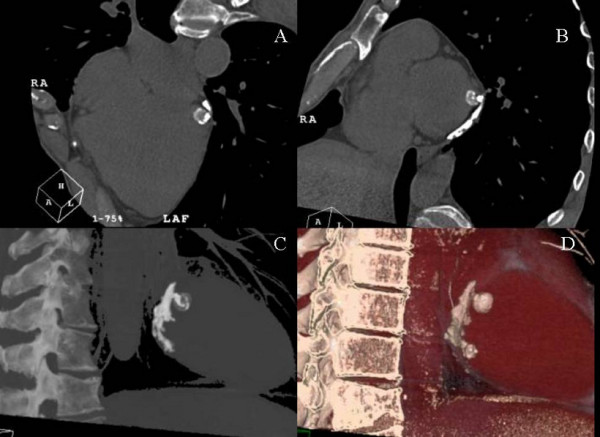
Patient 2: ECG-triggered 64-slices CT scan. Volume rendering images. Panel A: horizontal long axis. Panel B: short axis at the mitral annulus level. Panel C and D: the caseous calcification involves the postero-lateral mitral annulus and shows inhomogeneous attenuation.

Patient 2. A 70-year-old male, who had undergone bone marrow transplantation for a follicular non-Hodgkin's lymphoma, was referred after transthoracic echocardiography had identified a hyperechogenic intramyocardial mass in the postero-lateral basal wall. CMR (Figure 2) and CT scan (Figure 3) confirmed the diagnosis of caseous calcification of the mitral annulus.

Both patients were asymptomatic at the time of diagnosis and were treated conservatively.

## CMR features

The CMR findings of the two patients are similar. In T1-weighted (T1W) sequences (Panel A in Fig. 1 and 2) the masses are dark, and in fat suppressed T2-weighted (T2W) STIR sequences (Panel B in Fig. 1 and 2) they lack signal. The combination of dark T1W and T2W tissue signal is unusual for a cardiac mass [5] and suggests calcification. In balanced steady state free precession (bSSFP) images the regions of caseous calcification (* in Panel C in Fig. 1 and 2) appear only slightly darker than the normal myocardium, with a well-defined intramyocardial border. During first pass gadolinium contrast administration no enhancement can be observed (Panel E in Fig. 1 and 2). There was evidence of perfusion delay in the anterior mitral annulus (Fig. 1) and in the septal mitral annulus (Fig. 2), suggesting local extension of the disease process. Post-contrast T1-weighted sequence (Panel D in Fig. 1) is negative for enhancement of the mass, but fibrous tissue seems to surround and delimitate the caseous core. Delayed enhancement sequences were obtained only in patient 2 (Panel D in Fig. 2); an enhanced fibrous cap was found to surround a central core that showed no contrast enhancement.

Apart from citations in two CT-based case reports [1,2] we are not aware of previous descriptions of the CMR features of caseous calcification of the mitral annulus. We believe the hallmarks of this condition to be low signal in both T1-W and T2-W sequences, both before and after contrast, associated with a slightly-darker-than-myocardium signal in SSFP sequences. Further examples need to be studied, but it may prove feasible to diagnose caseous calcification of the mitral annulus by CMR without the need for further CT examination.

**Table 1:** CMR appearances found in the reported cases of caseous calcification of the mitral annulus

Pre-contrast T1-W	Pre-contrast T2-W	Pre-contrast bSSFP	First pass perfusion	Delayed Enhancement
DARK	BLACK	Slightly darker than myocardium	Not perfused	Enhanced border surrounding a non-enhanced core
